# AI-Guided Systems Neurogenomics in Neurodevelopmental Disorders

**DOI:** 10.3390/genes17091026

**Published:** 2026-08-28

**Authors:** Himanshu Goel, Tracy Dudding-Byth, Benjamin Kamien

**Affiliations:** 1 Hunter Genetics, Waratah, NSW 2298, Australia; tracy.dudding@health.nsw.gov.au; 2 The School of Medicine and Public Health, University of Newcastle, Newcastle, NSW 2308, Australia; 3 Genetic Health Western Australia, Perth, WA 6008, Australia

**Keywords:** artificial intelligence, systems neurogenomics, neurodevelopmental disorders, chromatinopathies, DNA methylation episignatures, regulatory network instability, developmental buffering, instability twins, digital neurodevelopmental twins, single-cell transcriptomics, multimodal AI, precision neurogenomics

## Abstract

Despite substantial advances in genomic testing, many individuals with neurodevelopmental disorders remain without a molecular diagnosis, while others receive a genetic diagnosis that does not fully explain phenotypic variability, developmental trajectory or tissue-specific consequences. Artificial intelligence (AI)-assisted methods are increasingly used for phenotyping, variant prioritisation, splice prediction, protein modelling, DNA methylation episignature classification and multi-omic analysis. However, these approaches differ substantially in evidentiary status and are often applied as separate prediction tasks rather than as components of an explicit mechanistic model. In this targeted narrative review, focused primarily on rare and genetically enriched neurodevelopmental disorders, we examine how AI-assisted methods may contribute to systems-level interpretation while remaining anchored to established molecular diagnosis and variant-classification frameworks. We propose a hypothesis-generating load-capacity framework comprising regulatory load, network capacity, developmental buffering and regulatory network instability. These are treated as operationalisable but currently unvalidated constructs. Regulatory instability is distinguished from stable disease-associated dysregulation, and threshold-like behaviour is presented as an empirical possibility rather than an assumed property of neurodevelopmental disease. We formulate five falsifiable predictions, consider how genomic, transcriptomic, epigenomic, single-cell, spatial, imaging, neurophysiological and longitudinal phenotypic evidence can provide complementary mechanistic constraints, and outline an auditable workflow following nondiagnostic genomic testing. We distinguish clinically implemented approaches from translational, emerging and conceptual applications, and emphasise calibration, evidence traceability, domain validity, prospective validation and appropriate abstention. Finally, we describe the Instability Twin as a prospective architecture composed of independently testable patient-specific sub-models rather than an existing clinical platform. The central proposition is that systems neurogenomics should be evaluated by whether mechanistically constrained integration provides reproducible information beyond established gene-level and simpler multimodal approaches.

## 1. Introduction

Neurodevelopmental disorders (NDDs), including intellectual disability, autism spectrum disorder and developmental epilepsies, are genetically and phenotypically heterogeneous. Genome-wide testing has substantially improved molecular diagnosis, yet a considerable proportion of affected individuals remain undiagnosed, and even after a pathogenic variant is identified, uncertainty may remain regarding phenotypic variability, developmental trajectory, tissue specificity, prognosis and potential therapeutic responsiveness [1,2,3,4,5,6,7,8,9,10,11,12,13,14,15].

Contemporary clinical genetics addresses these questions using substantially more than gene identification alone. Molecular mechanism, inheritance, allelic context, gene–disease validity, phenotype, segregation, functional evidence and variant-specific data are integrated to establish and refine causal diagnoses. For many rare NDDs, identification of a pathogenic or likely pathogenic variant within a well-established gene–disease relationship provides a robust molecular explanation and should remain the foundation of interpretation. A systems approach should therefore extend rather than replace molecular and gene-level diagnosis.

The rationale for such an extension arises from the developmental context in which genetic perturbations act. Neurodevelopment depends on coordinated chromatin regulation, transcription, RNA processing, signalling, cellular differentiation, migration, synaptic maturation and activity-dependent plasticity [15,16,17,18,19,20,21,22]. The consequences of a pathogenic perturbation may therefore depend not only on its primary molecular effect but also on the cell type, transcript, developmental stage and regulatory context in which that effect occurs. These contextual dependencies provide plausible routes through which similar primary molecular perturbations may produce different downstream consequences, or molecularly distinct disorders may converge on overlapping phenotypes.

Systems neurogenomics seeks to investigate these relationships by connecting genomic variation with molecular, cellular, developmental and clinical measurements. Importantly, this is not equivalent to simply increasing the number of data modalities analysed. A multimodal model that combines genomic, transcriptomic, epigenomic, imaging and phenotypic features without specifying how the layers are biologically related may improve prediction yet remain mechanistically uninformative. The systems objective is instead to ask whether cross-scale observations provide independent constraints on an explicit biological hypothesis.

AI-assisted methods may facilitate this task. Machine learning and foundation model approaches are now applied to structured phenotyping, facial analysis, variant prioritisation, splice prediction, protein structure, regulatory-sequence interpretation, DNA methylation classification, single-cell data and multimodal integration [23,24,25,26,27,28,29,30,31]. Their utility should not be assumed simply because the underlying data are high-dimensional. Conventional statistical approaches, network analysis, mechanistic modelling and experimental validation remain essential comparators. AI is therefore treated in this review as an enabling methodology whose value depends on whether it improves a defined interpretive, predictive or clinical task beyond simpler alternatives.

We focus primarily on rare and genetically enriched NDDs, where an identifiable or strongly suspected genomic perturbation can anchor downstream mechanistic investigation. Common neurodevelopmental and psychiatric traits with highly polygenic architectures raise related systems questions, but the relationship between distributed common-variant liability and the constructs developed here requires separate empirical evaluation. We therefore do not assume that a framework developed around identifiable rare disease perturbations generalises directly to common polygenic disease.

Within this domain, we propose a hypothesis-generating load-capacity framework. Regulatory load describes the effective perturbational burden imposed by a molecular alteration within a defined biological context. Network capacity refers to properties of the recipient system that may constrain propagation of that perturbation. Developmental buffering denotes the observed attenuation of perturbational effects sufficiently to preserve an expected developmental state or trajectory. Regulatory network instability describes reduced fidelity of the maintenance, transition or recovery of a regulatory or cellular state. These constructs are not presented as established quantitative variables or as replacements for existing concepts of robustness, canalisation, resilience, liability-threshold models, network controllability or attractor dynamics. Their proposed contribution is an operational translational scaffold linking patient-specific genomic perturbation with measurable downstream consequences.

A central distinction is that dysregulation is not equivalent to instability. A pathogenic variant may produce a stable abnormal expression state, altered dosage or reproducibly shifted developmental trajectory without generating dynamically unstable behaviour. Similarly, a nonlinear genotype–phenotype relationship does not establish the existence of a discrete biological threshold. Regulatory instability and threshold-like behaviour are therefore treated as testable possibilities rather than assumptions of the framework.

The novelty proposed here is not a new principle of biological robustness or network dynamics, but a testable translational architecture connecting genomic perturbation to measurable regulatory, developmental and clinical consequences and asking whether that integration provides information beyond established approaches.

### Scope and Literature Selection

This article is a targeted narrative review rather than a systematic review or meta-analysis. Its primary scope is rare and genetically enriched neurodevelopmental disorders, with emphasis on approaches that connect genomic variation to molecular, cellular and developmental mechanisms and on the potential role of AI in integrating these evidence layers.

During revision, a targeted literature update was conducted in PubMed/MEDLINE through 15 August 2026. Searches combined terms relating to neurodevelopmental disorders and rare disease genomics with variant interpretation, artificial intelligence and machine learning, regulatory genomics, RNA sequencing and splicing, DNA methylation episignatures, single-cell and spatial transcriptomics, computational phenotyping, neuroimaging, neurophysiology, multimodal integration and patient-specific or digital-twin modelling. Searches were supplemented by reference chaining from relevant reviews, consensus documents and primary studies, and by verification of recently published studies on publisher or PubMed records.

Literature was selected for relevance to the mechanistic or translational questions addressed in this review. Priority was given to primary methodological and validation studies, clinical implementation studies, relevant professional guidance and consensus frameworks, and recent studies that materially changed the capabilities or limitations of the field. Because the purpose is conceptual synthesis rather than exhaustive evidence enumeration, study identification and inclusion were not performed according to a systematic-review protocol and no claim of comprehensive literature capture is made. Where evidence is limited to retrospective studies, proof-of-concept experiments, preprints or adjacent disease domains, this is identified explicitly.

**Figure 1:**
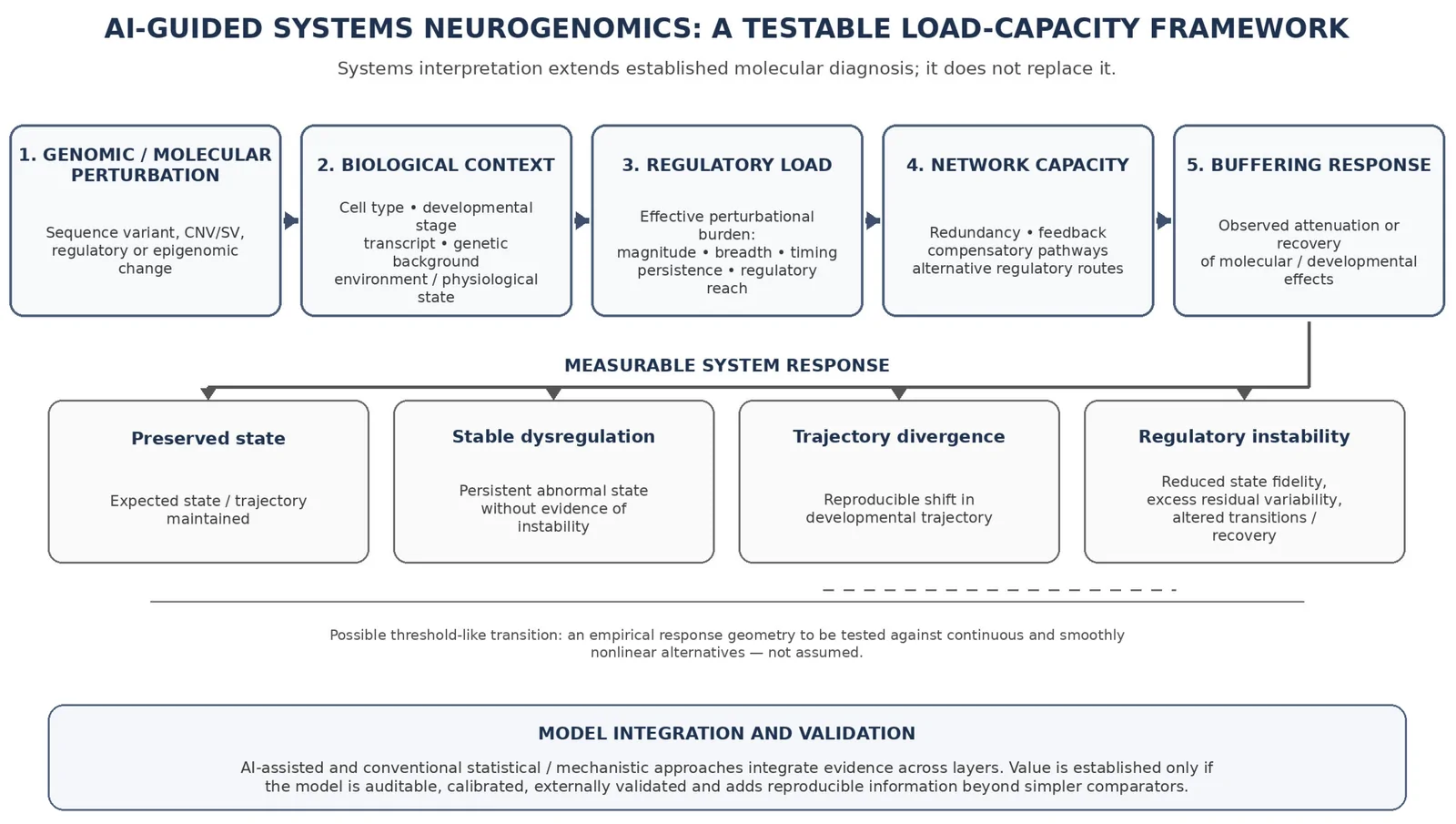
Proposed systems-neurogenomics load-capacity framework. A genomic or molecular perturbation acts within a context defined by cell type, developmental stage, regulatory architecture and broader genetic, environmental and physiological influences. Regulatory load represents effective perturbational burden, whereas network capacity represents properties that may constrain propagation of that perturbation. Developmental buffering refers to the observed attenuation or recovery of perturbational effects. Possible downstream responses include preservation of state, stable dysregulation, developmental trajectory divergence or regulatory instability. Regulatory instability is therefore one possible outcome rather than an assumed consequence of pathogenic variation. Threshold-like transitions may occur in some systems but are treated as an empirical possibility rather than a defining property of the framework. AI-assisted and conventional computational approaches can integrate measurements across these layers, but their value depends on the availability of reproducible information beyond simple mechanistic or statistical models.

Figure 1 and Table 1 summarise the operational vocabulary of the proposed framework and distinguish its latent constructs from measurable proxies and limitations.

Accordingly, the framework is useful only if its constructs can be estimated independently of the outcomes they are intended to explain and if their integration demonstrates incremental explanatory or predictive value beyond established approaches.

## 2. Systems Neurogenomics: A Conceptual Framework

### 2.1. Extending Variant-Level Diagnosis to Systems-Level Interpretation

Contemporary clinical genetics already integrates molecular mechanism, inheritance, gene–disease validity, phenotype, segregation, functional evidence and allelic context. For many rare neurodevelopmental disorders, identification of a pathogenic variant provides a robust causal diagnosis and should remain the foundation of interpretation. A systems perspective is therefore intended to extend rather than replace established gene- and variant-level reasoning.

The additional question addressed by systems neurogenomics is how an identified or suspected molecular perturbation propagates through the developmental context in which the affected gene operates. Neurodevelopment depends on interacting chromatin, transcriptional, RNA-processing, signalling, cellular and circuit processes whose consequences vary by cell type and developmental stage. The same molecular alteration may therefore have different downstream consequences according to transcript usage, regulatory context, developmental timing, genetic background and compensatory biology.

This distinction is particularly relevant to rare and genetically enriched neurodevelopmental disorders in which a primary molecular perturbation can often be identified but determinants of variable expressivity, tissue specificity or developmental trajectory remain incompletely understood. Systems-level analysis can generate hypotheses about these downstream processes but should not dilute established causal information from a well-supported monogenic diagnosis.

AI-assisted methods may facilitate this analysis by integrating high-dimensional sequence, regulatory, transcriptomic, cellular and phenotypic data. AI is an enabling methodology rather than a defining requirement: statistical genetics, network biology, mechanistic modelling and experimental validation remain equally important. The relevant question is whether an integrative model provides information beyond established and simpler approaches, not whether it uses a particular computational architecture. Functional neurogenomics across model systems and integrative neurogenomic workflows in adjacent neurological disease illustrate what such an extension requires in practice [32,33].

### 2.2. Regulatory Load, Network Capacity and Developmental Buffering

We use regulatory load as a latent construct describing the effective perturbational burden imposed by a genomic or molecular alteration within a specified biological context. Candidate determinants include the magnitude and mechanism of the primary molecular effect, breadth of affected downstream targets, developmental timing and persistence, and the regulatory position of the affected gene or element. These features are context-dependent and should, where possible, be measured independently of clinical severity.

Network capacity refers to properties of the recipient biological system that may constrain propagation of that perturbation or permit maintenance of function. Candidate contributors include regulatory redundancy, compensatory pathway or paralogue activity, feedback architecture, alternative regulatory routes and other context-specific mechanisms of robustness. Capacity is therefore not a synonym for mild phenotype and should not be inferred retrospectively from outcome.

We distinguish network capacity from developmental buffering. Capacity describes properties that could confer robustness; buffering describes the observed attenuation of perturbational effects sufficiently to preserve an expected developmental state or trajectory. Molecular compensation observed at a single time point is more appropriately described as regulatory or molecular compensation unless preservation of a developmental process has been demonstrated.

These constructs are hypotheses rather than directly observed quantities. Their value depends on whether independently measured features of perturbation and the recipient system predict molecular or developmental outcomes not used in their definition. Experimental strategies could vary perturbational magnitude while holding genetic background relatively constant or examine the same perturbation across genetic backgrounds or after manipulation of a candidate compensatory pathway.

The framework does not assume a fixed mathematical relationship between load and capacity. System response can instead be treated as a context- and time-dependent function of perturbation, capacity and additional biological influences. Clinical severity remains an outcome against which independently estimated constructs are tested.

### 2.3. Regulatory Network Instability as a Specific Hypothesis

Disease-associated dysregulation is not synonymous with regulatory instability. Dysregulation describes deviation from an expected molecular or cellular state; instability requires evidence that the maintenance, transition or recovery of that state has itself become less stable.

A pathogenic variant may therefore produce a reproducible reduction in expression, a stable shift in cell identity or an abnormal developmental trajectory without generating regulatory network instability. Such findings may establish disease mechanism but do not, by themselves, support the instability hypothesis.

Candidate evidence for instability includes prespecified dynamical properties such as reduced cell-state fidelity, excess residual biological variability, altered transition probabilities, increased trajectory dispersion or impaired recovery following a controlled perturbation. The appropriate measure will differ according to the biological system and should be defined before examining clinical outcome.

Regulatory instability should consequently be regarded as one possible downstream response to perturbation, not as the presumed explanation for neurodevelopmental disease. Stable loss of function, altered dosage, gain-of-function or dominant-negative effects, developmental timing, modifier variation and environmental influences may explain phenotypic heterogeneity without requiring instability.

### 2.4. Transcriptional Variability as One Candidate Readout

Single-cell transcriptomics provides one potential route for testing regulatory instability, but transcriptional noise requires a narrower definition than total expression variance. Here, we use the term to denote residual biological cell-to-cell variability in gene expression after accounting, as far as possible, for technical variation, cell-type composition, developmental state and other relevant measured covariates.

This should be distinguished from technical noise, heterogeneous cell composition, expected stochastic transcriptional bursting, physiological state variation and dispersion caused by asynchronous developmental trajectories. An increase in raw variance therefore does not constitute evidence of regulatory instability.

A stronger test would ask whether disease-associated cells show reproducibly increased residual variability, impaired state fidelity or altered transition behaviour relative to appropriately matched controls after technical and biological confounders have been modelled. Longitudinal, isogenic or perturbational designs would provide stronger evidence than cross-sectional differences alone.

Transcriptional variability is only one possible instability readout. Depending on the system, relevant measures could include recovery following perturbation, stability of cell-state assignment, transition probabilities, trajectory divergence or physiological variability. The framework should therefore not be defined by any single assay.

### 2.5. Continuous, Nonlinear and Threshold-like Responses

The framework does not assume that perturbational effects are governed by a discrete instability threshold. Increasing perturbational load could produce an approximately continuous response, a smoothly nonlinear response or, in some systems, an identifiable transition beyond which a prespecified molecular or developmental outcome changes disproportionately.

Only the latter should be described as evidence of threshold-like behaviour. A categorical clinical diagnosis does not establish an underlying biological threshold, and a sigmoid exposure–response relationship does not necessarily identify a discrete change point. Evidence for threshold-like behaviour should require comparison with appropriate continuous and nonlinear alternative models and, ideally, replication of the inferred transition in independent data or experimental systems.

For this reason, we refer throughout to a load-capacity framework, with threshold-like behaviour treated as one testable possible response geometry rather than a defining feature.

### 2.6. Testable Predictions of the Framework

A useful framework should generate predictions that can be tested and, in principle, falsified. Box 1 specifies the dataset-level observations that would support or refute the principal claims of the load-capacity framework.

Box 1 **Testable predictions of the load-capacity framework.**

**Prediction 1—Residual transcriptional variability**. In disease-relevant cell populations, some regulatory disorders will show greater residual biological expression variability than appropriately matched controls after technical variation, cell composition and developmental state are accounted for. Support: replicated residual variability or state-instability signal in independent or isogenic data. Refutation: equivalent variability after adequate adjustment, or a stable mean-state shift that explains the phenotype without evidence of instability.

**Prediction 2—Network-position effects**. For perturbations of comparable primary molecular magnitude, independently defined network properties such as connectivity or regulatory reach will contribute additional predictive information about downstream molecular effects. Support: network features improve held-out prediction beyond variant-effect magnitude and established covariates. Refutation: no reproducible incremental information after appropriate adjustment.

**Prediction 3—Capacity modifies equivalent perturbations**. Independently measured compensatory features will modify the molecular or developmental consequences of an equivalent primary perturbation. Support: manipulation or natural variation in a candidate capacity mechanism changes the downstream effect while the initiating perturbation is held constant. Refutation: candidate capacity measures neither predict nor experimentally modify the downstream response.

**Prediction 4—Threshold-like behaviour is demonstrable only in defined contexts**. Some perturbational systems will be better described by an identifiable nonlinear transition than by continuous alternatives. Support: a prespecified change-point or threshold model demonstrates reproducibly superior fit and predictive performance. Refutation: continuous or smoothly nonlinear alternatives perform equally well or better.

**Prediction 5—Mechanistically constrained integration adds incremental value**. Integration of genomic, molecular, cellular and phenotypic information through explicit mechanistic constraints will improve a prespecified explanatory or predictive task beyond established gene-level, unimodal or unconstrained multimodal models. Support: reproducible improvement in held-out or prospective data. Refutation: no incremental performance, calibration or decision utility relative to simpler comparators.

These predictions establish a standard against which the framework should be evaluated: constructs should be independently operationalised, competing models should be compared explicitly, and multiscale integration should demonstrate incremental value rather than merely increasing model complexity [34,35,36].

### 2.7. Core AI Methodologies

AI methods used in neurogenomics range from classical supervised and unsupervised learning to deep sequence models and foundation models. Their relevance depends on the task. Supervised classifiers underpin variant prioritisation, episignature assignment and phenotype recognition; unsupervised and self-supervised approaches identify structure in high-dimensional molecular data; sequence models predict splicing, missense and regulatory effects; and foundation models transfer representations learned from large biological corpora to smaller disease-specific datasets.

None of these architectures is intrinsically mechanistic or clinically valid. Dimensionality reduction can reveal visually separated clusters without establishing diagnostic boundaries; attention weights or feature attribution do not establish causality; and representations learned from population-scale datasets may be miscalibrated for rare constitutional disease. Evaluation should therefore be tied to the intended use, relevant comparator, calibration and external validation rather than to model class alone.

For this review, clinical/near-clinical denotes methods validated for a defined diagnostic or interpretive use; translational denotes substantial supporting evidence without broad prospective validation; emerging denotes early or restricted-dataset applications; and conceptual denotes proposed architectures without patient-level validation. Readiness is assigned to an intended use, not to a technology in the abstract. Table 2 summarises the principal AI methodologies applied across neurogenomics, with their typical inputs, outputs and representative tools.

## 3. AI-Assisted Genomic Variant Interpretation

### 3.1. Variant Prioritisation

AI-assisted variant interpretation is most useful when it narrows a clinically testable hypothesis rather than producing an autonomous classification. Sequence-level predictors, population constraint, phenotype matching and regulatory models can prioritise candidates, but their outputs should remain anchored to established gene–disease validity and ACMG/AMP- and ClinGen-aligned evidence frameworks. A practical sequence is: computational prioritisation; formulation of a biological hypothesis; selection of mechanism-appropriate orthogonal evidence; and only then integrated variant classification, counselling and management. REVEL, CADD, AlphaMissense and gene-level constraint metrics can contribute at the first stages, but none independently establishes pathogenicity or mechanism [45,46,47,48,49,64,65,66,67,68,69,70,71,72,73,74].

AlphaMissense integrates evolutionary and structural information through protein language modelling to provide proteome-wide estimates of missense variant effect [45,46,47,48,49]. A high score supports a computational hypothesis that a substitution may be functionally consequential; it does not confirm protein damage or distinguish loss of function, gain of function, dominant-negative action, altered localisation or disrupted protein interaction. popEVE similarly combines evolutionary and population information to rank missense variation [49,73,74]. These tools are therefore best used to prioritise variants and identify plausible mechanisms for subsequent evaluation, not to determine mechanism from score magnitude alone.

Regulatory variant interpretation is becoming increasingly important as clinical sequencing moves beyond coding regions. AlphaGenome predicts multiple functional genomic outputs, including gene expression, chromatin accessibility, histone modifications, transcription-factor binding, chromatin contacts and splicing signals, across long sequence contexts from primary sequence [43,44,75,76]. Sequence-to-function models provide a scalable means of generating hypotheses about noncoding variant effects, but these predictions remain computational evidence. Patient-specific and brain-relevant validation is limited, and predictions should be tested in the relevant gene, tissue, cell type and disease context before contributing to a clinical mechanistic conclusion.

Across AI-derived outputs, four stages should remain distinct: hypothesis prioritisation; supporting computational evidence; demonstrated molecular consequence; and integrated clinical classification or management. Population constraint provides gene-level context rather than variant-level proof; phenotype matching supports gene–disease concordance but depends on specificity and completeness; splice and regulatory predictions generate testable hypotheses; and experimental or molecular assays contribute evidence only within their validated mechanism and domain. The clinically relevant question is whether independent evidence streams converge on a mechanism under an appropriate classification framework, not whether several correlated models assign high scores. Table 3 lists representative tools and approaches across each layer of the interpretive workflow, with their clinical role, principal limitation and readiness level.

### 3.2. AI and RNA/Splicing Prediction

Splicing disruption is a major and systematically underdetected mechanism in genetic disease. Many pathogenic splice-altering variants lie outside canonical donor and acceptor dinucleotides or are annotated as synonymous, missense or intronic. Deep sequence models such as SpliceAI, Pangolin, AbSplice and FexSplice expand the set of variants that can be prioritised for transcript-level testing [25,27,28,77,78,79].

Splice prediction should be interpreted as a hypothesis about transcript consequence. The relevant clinical question is not simply whether a score exceeds a threshold, but whether the predicted splice event affects a disease-relevant transcript, whether that transcript is expressed in the assayed tissue, and whether the predicted consequence is compatible with the established gene–disease mechanism. Computational splicing evidence should therefore be calibrated and applied within appropriate ACMG/AMP and ClinGen specifications rather than treated as a standalone diagnostic output [28,111].

RNA sequencing can directly observe transcript consequences, including exon skipping, cryptic splice-site activation, intron retention, altered abundance and allele-specific expression. Tissue relevance is critical: a negative result in blood may be non-informative for a brain-specific exon or developmentally restricted transcript. Patient-derived cells, induced pluripotent stem-cell models or organoids can provide research substrates when accessible tissue does not express the relevant isoform, but their model-system limitations must also be considered [25,26,27,28,29,30,31].

Observed RNA abnormalities should not automatically be labelled functional validation or mapped generically to PS3. ClinGen SVI guidance recommends using an appropriately weighted PVS1_Strength (RNA) framework when RNA studies demonstrate a loss-of-function transcript consequence, whereas PS3/BS3 is reserved for well-established assays measuring functional impact not already captured by the splicing result [111]. Expression outliers, allele-specific expression and other transcriptomic abnormalities can be mechanistically informative but require gene-, assay- and disease-specific evaluation before assignment of ACMG/AMP evidence strength.

### 3.3. Structural Neurogenomics

Protein structure prediction has become a useful source of mechanistic context for missense variant interpretation. AlphaFold and related approaches can identify domain organisation, candidate interfaces and structural environments at proteome scale, while protein language models capture evolutionary constraints not evident from structure alone [80,81,82,83,84,85,86,87]. Predicted structures are less reliable for intrinsically disordered regions, conformational changes, context-dependent complexes and direct inference of functional consequence, and high structural confidence should not be confused with pathogenicity evidence.

Structural context can refine hypotheses when disease mechanisms are domain specific, for example variants affecting ion-channel pores, DNA-binding domains, chromatin-reader modules, ATPase cassettes or protein–protein interfaces. Its role is to constrain which mechanisms are plausible and which functional assays are most informative; it does not establish the direction of effect or in vivo disease mechanism.

A predicted destabilising effect may be compatible with loss of function but cannot by itself distinguish haploinsufficiency from dominant-negative action. Likewise, a predicted interface disruption does not establish that the relevant interaction is disease limiting. Structural evidence should therefore direct functional testing and be integrated with inheritance, phenotype, gene–disease mechanism and orthogonal data rather than used as an autonomous classification criterion.

Accordingly, structural modelling is best treated as mechanistic contextualisation. A structurally plausible explanation may strengthen biological coherence, but clinical classification requires evidence categories with defined validity and independence under the applicable interpretation framework.

### 3.4. Challenges in Variant Interpretation

Neurodevelopmental variant interpretation remains difficult because many disease genes have direction-sensitive mechanisms, developmentally restricted effects or cell-type-specific consequences. Penetrance and expressivity can also reflect modifier variation, polygenic background, mosaicism and environmental context. AI predictions are therefore structured evidence components that narrow the hypothesis space and direct testing, not standalone diagnostic outputs. Clinical interpretation still requires segregation, population data, phenotype, gene–disease validity, mechanism-specific functional evidence and expert synthesis [40,113,114].

**Figure 2:**
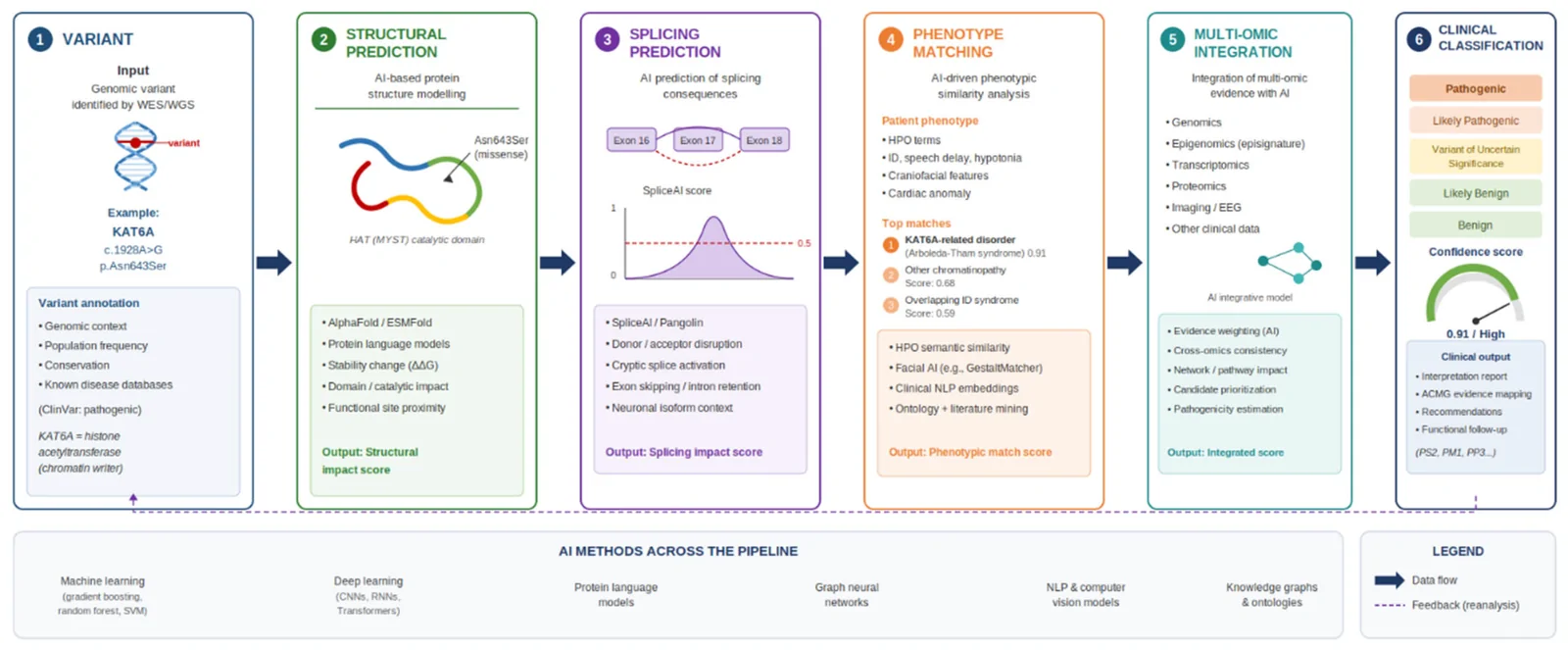
AI-assisted variant interpretation pipeline. AI-supported analyses can prioritise a candidate and define a mechanistic hypothesis through variant annotation, structural prediction, splicing prediction, phenotype matching and multi-omic integration. These outputs feed into evidence integration rather than directly into a diagnosis: computational predictions are interpreted within ACMG/AMP- and ClinGen-aligned frameworks and, where required, tested using orthogonal functional assays before clinical classification, counselling or management. The workflow is illustrated with the pathogenic *KAT6A* variant c.1928A > G; p.(Asn643Ser) (Arboleda-Tham syndrome). WES, whole-exome sequencing; WGS, whole-genome sequencing. ClinVar, Clinical Variation; AL, Artificial Intelligence; ESM, Evolutionary Scale Modeling; HPO, Human Phenome Ontology; ID, intellectual disability; NLP, Natural Language Processing; ACMG, American College of Medical Genetics and Genomics; SVM, Support Vector Machine; CNN, Convolutional Neural Network; RNN, Recurrent Neural Network.

The same principle applies across other modalities. A validated episignature may provide important molecular support but its evidentiary weight depends on disorder, variant mechanism, tissue, mosaicism, assay validation and classifier domain. Phenotype-matching systems support candidate ranking but are sensitive to coding quality, age and ascertainment. AI-derived evidence is most clinically useful when it performs one of three explicit functions: prioritising a candidate, defining a testable mechanism or selecting the most informative next assay. Figure 2 shows where these outputs enter the interpretive pipeline and where confirmatory evidence is required.

## 4. AI-Assisted Phenomics and Rare Disease Diagnosis

### 4.1. Human Phenotype Ontology and Computational Phenotyping

The Human Phenotype Ontology (HPO) provides a structured, computable vocabulary for clinical features and enables systematic comparison between patients, genes and disease profiles [50,51,52,53,60,63]. HPO-driven prioritisation tools improve candidate variant ranking by matching a patient’s phenotype to known gene–disease associations, a particularly valuable function when sequencing identifies multiple plausible candidates and the phenotype must arbitrate between them. The diagnostic yield of sequencing is therefore not independent of phenotype quality: a precisely coded HPO profile improves prioritisation, while a coarse or incomplete one dilutes it [55,56,58,61,63,64,65]. Automated HPO extraction is becoming operationally important as genomic medicine scales beyond what manual annotation can sustain. FastHPOCR, PhenoBERT, PhenoTagger and PhenoRerank represent different approaches to phenotype concept recognition, structured annotation and candidate re-ranking from clinical text [55,56,58,60,62]. These systems can reduce annotation burden and support cohort-level phenotype discovery, but they must handle the linguistic complexity of clinical documentation accurately. Negation, uncertainty, temporality and differential diagnosis framing each require specific handling; the distinction between “no regression” and regression, or between “query seizures” and confirmed epilepsy, is clinically critical and must be preserved rather than collapsed by an annotation model [55,56,58,60,62].

Phenotype ontologies can also be linked to other biological knowledge frameworks. HPO2GO connects phenotype terms to gene ontology associations, enabling inference from clinical features to molecular function and cellular process [63]. MitoPhen demonstrates disease-specific HPO application for identifying mitochondrial DNA disease patterns from phenotypic signatures [94]. These connections are relevant to systems neurogenomics because they provide a structured path from bedside observation to pathway, cellular mechanism and regulatory network, the direction of inference that systems-level interpretation requires.

### 4.2. Facial AI and Dysmorphology and the FaceMatch Platform

Facial AI systems use computer-vision models to identify syndrome-associated craniofacial patterns from photographs and can provide a phenotypic signal complementary to genomic and clinical data. GestaltMatcher, Face2Gene/DeepGestalt and related systems embed facial features in learned phenotypic spaces that can support syndrome prioritisation and rare disease matching [38,39,40,41,42]. Their appropriate role is phenotype support and hypothesis generation rather than autonomous diagnosis.

Facial morphology varies with ancestry, age, sex, image quality and ascertainment, and some NDDs have no consistent facial gestalt. Clinical or research use therefore requires explicit consent and governance for image capture, storage, secondary use and model development; attention to re-identification risk; transparent reporting of ancestry and demographic performance; and caution when a patient falls outside the training domain [38,39,42,95,96,112]. Longitudinal platforms such as FaceMatch can improve age-aware representation and reverse phenotyping, but prospective validation across diverse populations remains necessary before performance can be assumed to generalise.

### 4.3. Multimodal Diagnosis and Cross-Scale Constraint

Multimodal integration is most informative when each modality constrains a different biological scale. A useful sequence is molecular perturbation → cell type and developmental window → structural network substrate → dynamic circuit behaviour → longitudinal clinical phenotype. Genomic and molecular data anchor the initiating mechanism; single-cell and spatial references identify plausible vulnerable populations; structural MRI and tractography describe anatomy and connectivity; EEG/MEG provide dynamic neurophysiological measurements; and longitudinal phenotype tests whether the resulting model is consistent with the patient over time [54,89,115].

Diffusion MRI and tractography should be interpreted as estimates of structural connectivity rather than direct measurements of axonal conduction velocity. In adjacent neurological domains, patient-specific virtual-brain models have combined diffusion imaging with MEG to infer conduction parameters and relate them to clinical impairment, providing proof-of-principle for model-derived physiological parameters calibrated against neurophysiology [116,117]. These studies support a cross-scale modelling strategy but do not establish equivalent validity in rare developmental disorders.

EEG- and MEG-derived network measures are also state-dependent. Sleep deprivation and menstrual-cycle phase can alter functional-connectivity or dynamic-network measures in healthy participants [113,118], and medication exposure, seizure proximity, fatigue, developmental stage and other physiological states may introduce additional variation. Candidate instability biomarkers therefore require state annotation, repeated measurements where feasible, and explicit covariate control. Across multimodal models, uncertainty should be preserved rather than averaged away, and discordant modalities should trigger review of input quality, annotation, model domain and biological assumptions before mechanistic interpretation.

### 4.4. Deep Phenotyping in Neurodevelopmental Disorders

Deep phenotyping moves beyond broad diagnostic labels (autism, epilepsy, developmental delay) to capture the structured dimensions that carry biological meaning: onset timing, developmental trajectory, regression, seizure semiology, seizure type and frequency, epilepsy syndrome classification, ictal and interictal EEG features, pharmacological treatment response and pharmacoresistance, motor pattern, sleep architecture, behavioural profile, growth parameters, autonomic features, neuroimaging findings and neurophysiology [7,40,114,119,120]. AI model performance in genomic interpretation is directly dependent on phenotype quality. Coarse phenotyping produces coarse prioritisation regardless of model sophistication: the genomic interpretation is only as precise as the phenotypic input that guides it. Epilepsy phenotyping deserves particular emphasis. Seizure onset age, semiology, syndrome classification and EEG signature carry diagnostic and mechanistic information that broad labels obscure. Infantile epileptic spasms syndrome, Dravet syndrome, *KCNQ2*-related neonatal epilepsy and glucose transporter type 1 deficiency syndrome each have characteristic electroclinical signatures that, when structured and computable, direct genomic interpretation toward specific gene classes, molecular mechanisms and treatment pathways. The distinction between a sodium channel gain-of-function epilepsy (responsive to low-dose sodium channel blockade) and a GABAergic interneuron disorder requiring a fundamentally different approach is not recoverable from the label “epileptic encephalopathy” alone. It requires structured electroclinical phenotyping integrated with genomic and functional data. The same requirement now extends before birth, where structured prenatal phenotyping and foetal phenotype ontology work are increasingly necessary for interpreting prenatally identified variants [121,122].

Deep phenotyping is also the primary tool for understanding variable expressivity. Patients carrying variants in the same gene may differ substantially in epilepsy severity, seizure type, treatment response, movement phenotype, language trajectory, autonomic dysfunction and psychiatric features. Without structured longitudinal phenotype data, these differences collapse into the same diagnostic label and their biological determinants, modifier variants, epigenomic context, sex, and stochastic developmental variation become invisible to analysis. Structured longitudinal phenotypes allow AI systems to learn developmental trajectories rather than static cross-sectional states, which is the appropriate unit of analysis for neurodevelopmental disease. Early hypotonia followed by epilepsy onset and cognitive regression carries different biological implications from stable developmental delay without seizures, or from epilepsy with preserved cognition and spontaneous remission, even when all three presentations receive the same broad diagnostic classification. Capturing this trajectory structure is not a refinement of phenotyping practice; it is a prerequisite for mechanistic interpretation.

## 5. AI in Epigenomics and Chromatin Disorders

### 5.1. Epigenomic Regulation in Neurodevelopment

Epigenomic regulation is integral to neurodevelopment. DNA methylation, histone modification, chromatin accessibility, enhancer–promoter communication and three-dimensional genome organisation contribute to the gene-expression programmes required for neural progenitor specification, neuronal differentiation, synaptic maturation and circuit formation. Disruption of these processes underlies many chromatinopathies and other regulatory NDDs [97,98,99].

Epigenomic assays can provide molecular readouts of regulatory consequences that are not captured by sequence annotation alone. Disease-associated methylation signatures are distributed, high-dimensional patterns, making supervised classification a natural analytical approach. Their clinical meaning, however, depends on reference-cohort quality, assay validation, ancestry and age representation, tissue, cell composition and the specific disorder being tested [97,98,99,100]. A disorder-associated episignature is a biomarker pattern; it should not itself be described as pathogenic.

### 5.2. DNA Methylation Episignatures in Diagnosis and Variant Interpretation

DNA methylation episignatures are reproducible genome-wide methylation patterns associated with an expanding subset of neurodevelopmental and chromatin disorders [97,98,99,100,101,102,112]. When assessed using a clinically validated classifier within its established disease and tissue domain, a concordant episignature can provide molecular evidence supporting a suspected diagnosis and may contribute to interpretation of variants of uncertain significance. In some sequencing-negative individuals, matching to a validated signature can direct additional genomic or clinical investigation toward a specific disorder. The result should nevertheless be interpreted as one component of the diagnostic evidence rather than as an assay-independent determination of pathogenicity.

**Figure 3:**
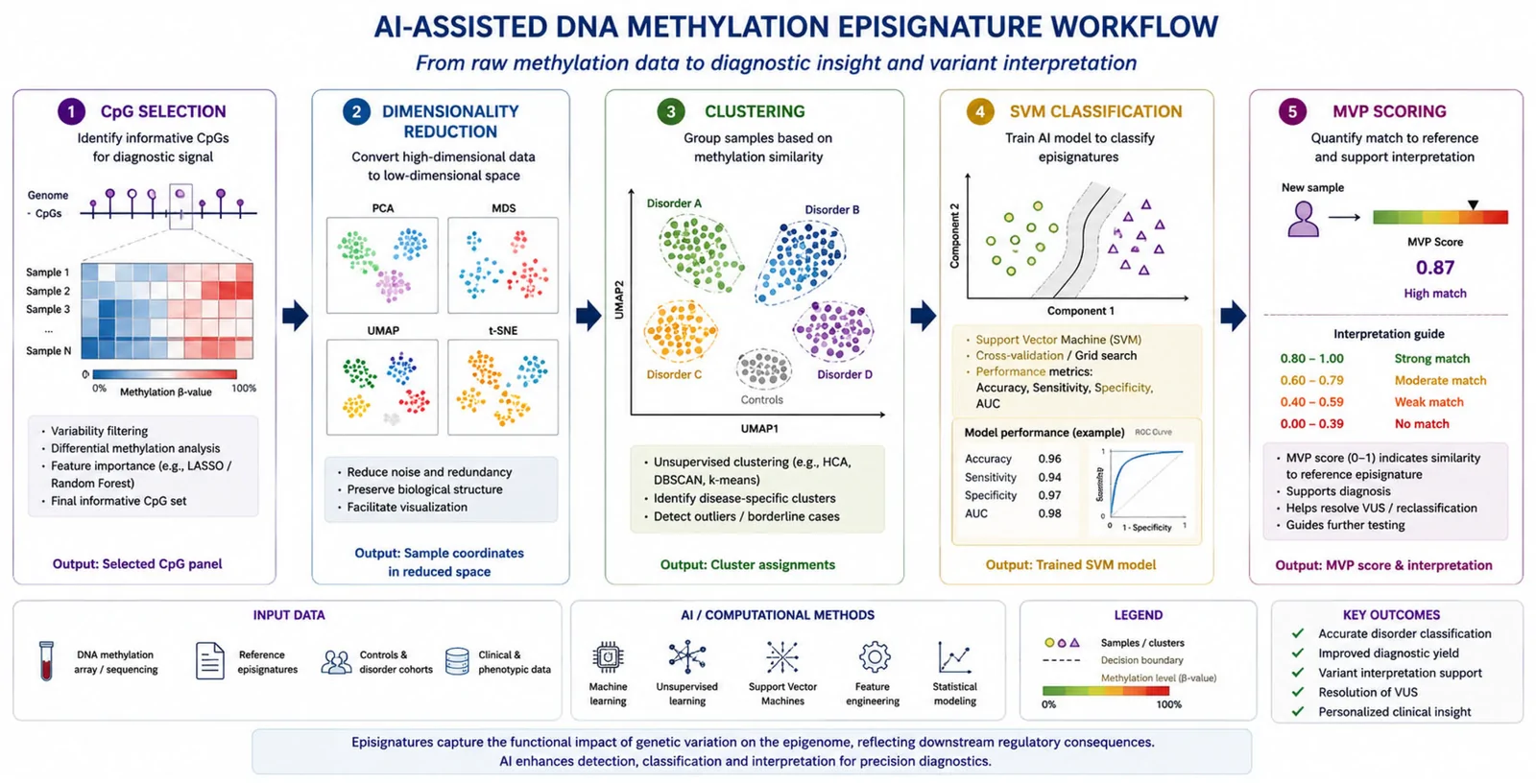
AI-assisted DNA methylation episignature workflow. Informative CpGs are selected from genome-wide methylation data, visualised using dimensionality-reduction methods and interpreted with clustering and supervised classifiers. Classifier scores can provide molecular support for diagnosis and selected VUS interpretation within validated disease and tissue domains, but require appropriate controls, comparator disorders, batch assessment and clinical context. Lasso, Least Absolute Shrinkage and Selection Operator; PSA, Principal Component Analysis; MDS, Multidimensional Scaling; UMAP, Uniform Manifold Approximation and Projection; HCA, Hierarchical Cluster Analysis; DBSCAN, Density-Based Spatial Clustering of Applications with Noise; SVM, Support Vector Machine; AUC, Area Under the Curve; MVP, Methylation Variant Pathogenicity; VUS, Variant of uncertain significance.

For variant interpretation, the relevant question is whether the methylation assay measures a biological consequence sufficiently linked to the established disease mechanism and whether the classifier has been adequately validated using appropriate pathogenic, benign and disease-comparator controls. A concordant result may then contribute functional evidence at a strength appropriate to that validation; it should not automatically be equated with PS3 or with a pathogenic classification [123]. Negative or borderline results also require caution: absence of a blood episignature may reflect limited classifier sensitivity, variant-mechanism differences, age or cell composition, technical factors or absence of a peripheral signature for the disorder. Blood methylation is therefore a peripheral molecular biomarker, not a direct measurement of regulatory state in the developing brain. Sensitivity, specificity, comparator set and domain boundaries should accompany interpretation. Three result categories therefore warrant separate reporting: a negative result in a patient with a strong clinical prior is uninformative rather than exculpatory; a borderline score should be reported with the classifier threshold and the distance from it; and a result obtained outside the validated ancestry, age, tissue or comorbidity range should be reported as uninterpretable rather than negative. Where the clinical prior remains strong, complementary neural-lineage substrates such as olfactory neuroepithelium, patient-derived neuronal or organoid models, and emerging single-cell brain methylation atlases can be pursued as functional investigation rather than as substitute assays. Figure 3 outlines the episignature workflow from CpG selection to classifier output and domain-aware interpretation.

### 5.3. Chromatinopathies as Systems Disorders

Chromatinopathies illustrate why a systems extension can be useful without displacing monogenic causality. A pathogenic variant in a chromatin regulator can alter many downstream targets across cell types and developmental stages, producing distributed transcriptional and epigenomic consequences. Overlapping clinical phenotypes across distinct chromatin disorders may therefore reflect convergence on shared developmental programmes, but this convergence does not by itself establish regulatory instability.

Direction-sensitive phenotypes further emphasise the need to retain molecular mechanism. Loss- and gain-of-function perturbations of related regulatory processes can produce overlapping or contrasting somatic and neurological outcomes [107]. These observations are compatible with context-dependent robustness and compensation, but they should not be used to infer a universal optimal regulatory equilibrium or a threshold without direct evidence.

Variable expressivity may reflect the interaction of the primary variant with modifier alleles, broader rare-variant or copy-number burden, polygenic liability, mosaicism, developmental timing and other biological or environmental context. Within the proposed framework, these factors are candidate determinants of perturbational load or recipient-system capacity that require independent measurement rather than retrospective assignment from phenotype severity.

ARID1B-related disorder provides an example of a genetically defined chromatinopathy with distributed downstream effects and a recognised methylation signature [97,112]. Such findings support system-level regulatory disturbance but do not demonstrate that clinical severity is determined by a load-capacity threshold or by regulatory instability. Those propositions require direct comparison of independently measured molecular, cellular and developmental readouts.

### 5.4. AI and Chromatin-State Modelling

AI-assisted chromatin modelling can integrate methylation, histone modification, chromatin accessibility, regulatory topology and transcriptional output to generate hypotheses about regulatory consequences. EPInformer integrates promoter-enhancer sequence with multimodal epigenomic data, while AlphaGenome predicts multiple sequence-to-function outputs across long genomic contexts [43,44,75,76,89]. These approaches can prioritise candidate regulatory mechanisms, but their patient-specific and brain-developmental validity remains incomplete.

The interpretive strength of multimodal epigenomic analysis lies in convergence of partially independent observations, not in score stacking. Concordant predictions across regulatory layers can support a mechanistic hypothesis; discordance should trigger review of assay context, cell type, transcript, model domain and technical quality. Convergent dysregulation should not be relabelled regulatory instability unless dynamical instability has been measured using prespecified criteria.

AI-assisted chromatin modelling should therefore aim to specify which regulatory consequence is predicted, in which cell type and developmental window, and what orthogonal assay could strengthen or refute that hypothesis. Whether such integration adds clinically useful information beyond sequence interpretation and validated episignature testing is an empirical question.

## 6. AI in Transcriptomics and Single-Cell Neurogenomics

### 6.1. Single-Cell Transcriptomics and Brain Cell Atlases

Single-cell and single-nucleus transcriptomics resolve gene expression across cell populations that are obscured in bulk tissue. Brain cell atlases can therefore identify the cell types and developmental stages in which a candidate gene or pathway is most active and provide reference states against which disease models can be compared [15,124,125,126,127,128,129]. These data generate spatial and developmental priors; they do not by themselves establish that the most highly expressing cell type is the causal substrate of the clinical phenotype.

Foundation models such as Geneformer and atlas resources such as STAB2 and ZEBRA extend this approach by learning or cataloguing cellular-state structure across large datasets [124,125,126,127,128]. Translation remains limited by incomplete representation of rare transitional states, differences between post-mortem reference tissue and developing patient biology, batch and ancestry effects, and uncertainty about how learned representations map to causal mechanisms.

### 6.2. Spatial Transcriptomics and Anatomical Priors

Spatial transcriptomics preserves anatomical context while measuring gene expression, allowing molecular perturbations to be related to cortical layers, nuclei, local cellular neighbourhoods and projection systems [130,131]. This is particularly relevant when a broadly expressed gene produces anatomically selective vulnerability. Spatial data can nominate regions and cell populations in which the local regulatory environment makes a molecular perturbation more consequential.

A cross-scale model should then test these molecular priors against patient-level anatomy and physiology rather than assume that a spatial expression pattern predicts circuit dysfunction. A practical sequence is genotype or molecular perturbation → vulnerable cell type and developmental window → spatial prior → patient-specific structural network → dynamic neurophysiology → clinical trajectory. Each transition provides an opportunity for disconfirmation as well as support [120,121,122].

### 6.3. Developmental Trajectory Modelling

Developmental trajectory models ask when and how a disease-associated molecular or cellular state diverges from an appropriate reference trajectory. Patient-derived cells, induced pluripotent stem-cell models and cerebral organoids can provide experimental systems for testing such hypotheses, while RNA sequencing can identify aberrant splicing, allele-specific expression, cryptic exon inclusion, intron retention and nonsense-mediated decay [25,26,27,28,29,30,31]. Model-system fidelity, differentiation variability and incomplete correspondence to in vivo developmental timing remain major limitations.

Trajectory divergence may be continuous, smoothly nonlinear or, in some settings, threshold-like. Model architecture should therefore accommodate nonlinear and branching developmental processes without prespecifying a discrete failure threshold. Longitudinal or perturbational data are preferable to cross-sectional snapshots because they allow transition dynamics, recovery and state fidelity to be tested directly.

## 7. Multi-Omics Integration and Systems Neurogenomics

### 7.1. From Complementary Evidence to Mechanistic Synthesis

Each modality in neurogenomics has a distinct error structure and evidentiary role. Genomic data identify candidate causal variation but may leave mechanism unresolved; RNA can observe transcript consequences but is tissue and stage dependent; methylation assays can provide disorder-associated molecular signatures within validated domains; single-cell and spatial references nominate vulnerable cell types and developmental windows; imaging and neurophysiology constrain structural and dynamic circuit consequences; and phenotype anchors these findings to the patient. Integration should preserve these distinctions rather than collapse them into a single score.

A clinically useful synthesis is therefore hypothesis-centred. Computational outputs first define a candidate mechanism; the next modality is selected because it can strengthen or refute that mechanism; and the resulting evidence is interpreted through the appropriate clinical framework. Discordance is informative only after technical quality, annotation, transcript choice, phenotype coding, model domain and dependence between evidence sources have been audited. When these checks do not resolve conflict, the correct conclusion may be that the mechanism remains uncertain.

Mechanistically constrained integration should also be tested against simpler alternatives. If combining regulatory, cellular, imaging and phenotypic data does not improve calibration, prediction, explanatory discrimination or clinical decision utility beyond established gene-level or unimodal models, the additional complexity is not justified.

### 7.2. A Practical Workflow After Nondiagnostic Genomic Testing

The most immediate clinical application is not an autonomous systems model but a disciplined sequence for deciding what to do after nondiagnostic testing. The workflow begins with a technical audit of the original assay and updated phenotype because a negative result may reflect assay limitations or outdated knowledge rather than a failure of genomic diagnosis. Reanalysis is followed by mechanism-specific prediction and selective deployment of RNA, methylation, multimodal phenotyping or experimental assays according to the uncertainty that remains. Box 2 sets out this workflow as a sequence of steps for a case that remains undiagnosed after exome or genome sequencing.

Box 2 **Practical AI-enabled workflow after nondiagnostic exome or genome sequencing.**

Step 0—Audit the technical scope of prior testing. Confirm what the assay and pipeline could reliably detect, including CNVs, structural variants, repeat expansions, mitochondrial variants, mosaicism and noncoding variation. Reanalyse what the existing data can support; retest when the required variant class or tissue was not adequately assayed.

Step 1—Re-phenotype longitudinally. Recode the phenotype using current HPO terminology, including absent features, onset, progression, regression, treatment response and age-dependent findings.

Step 2—Reanalyse sequence data with current knowledge. Update gene–disease relationships, transcript annotations, inheritance-aware filters and relevant variant classes. Talos illustrates the scalability of iterative reanalysis: in 4735 previously undiagnosed individuals it identified 241 additional diagnoses (5.1%) [132].

Step 3—Apply mechanism-specific computational prediction. Use missense, splice, structural and regulatory models to generate explicit biological hypotheses rather than aggregate scores. Audit discordant outputs for input quality, transcript choice, phenotype coding, model domain and dependence between predictors.

Step 4—Use RNA when transcript mechanism is plausible. Select an informative tissue where possible and interpret observed splicing or expression consequences using mechanism-appropriate ClinGen guidance [111].

Step 5—Use episignature testing where validated and relevant. Interpret concordant, negative and borderline results within the specific classifier, tissue and disease domain; do not equate a positive signature automatically with a fixed ACMG/AMP criterion [123].

Step 6—Add multimodal phenotype data selectively. Use facial analysis, MRI, EEG/MEG and longitudinal developmental data when they resolve a defined uncertainty. Preserve state, technical and demographic limitations rather than treating more modalities as greater certainty.

Step 7—Escalate unresolved high-priority hypotheses experimentally. Patient-derived cells, organoids, single-cell assays or targeted functional studies can test specific mechanisms, particularly in novel gene–disease discovery.

Step 8—Report transparently. State the supported mechanism, input quality, model version and domain, uncertainty, evidence status, residual uncertainty and the next assay that would most efficiently strengthen or refute the interpretation.

The near-term objective is to improve the efficiency, transparency and mechanistic coherence of investigation. Whether the complete systems framework increases diagnostic yield, prognostic accuracy or patient-relevant outcomes beyond current practice remains an empirical question requiring prospective evaluation. Box 3 condenses the workflow into practice points for routine use.

Box 3 **Key practice points.**

- Start with phenotype quality and technical assay scope before adding new AI tools.
- Reanalysis is a recurring process: in Talos, iterative monthly review reduced the candidate burden to approximately one variant per 200 cases after initial analysis [132].
- Read computational scores as hypothesis-generating evidence, not verdicts; map each output to an appropriate ACMG/AMP- or ClinGen-aligned evidence pathway.
- Use RNA and episignatures only when the mechanism, tissue and validated assay domain make the result interpretable.
- Add modalities that resolve a specific uncertainty, preserve discordance and uncertainty, and escalate to experimental testing when a clinically important mechanism remains unresolved.

## 8. Limitations, Validation and Governance

### 8.1. Domain Validity, Representation and Dataset Shift

AI models are conditional on the populations, assays, phenotypes and technical environments in which they were developed. This limitation is especially important in rare neurodevelopmental disorders, where training cohorts are small, ancestry representation is uneven and individual syndromes may be represented by few examples. Performance observed within a development cohort should not be assumed to generalise across ancestry, age, disease severity, molecular mechanism, laboratory workflow or clinical setting.

Population-frequency resources, methylation classifiers, facial-analysis systems and phenotype models each have modality-specific sources of distribution shift. Validation should describe the populations and technical domains represented in development and testing and identify groups for which performance remains uncertain. Facial-analysis systems require particular caution because the input is identifiable and performance may vary with ancestry, age, sex, image quality and ascertainment. Appropriate consent, governance for secondary use, re-identification safeguards and demographic performance reporting are therefore part of technical validity rather than optional ethical add-ons.

The relevant question is not whether a model has a high global performance metric, but whether performance is characterised in a population and context sufficiently similar to the patient in whom it is being applied. Outputs outside that validated domain should be down-weighted, explicitly qualified or withheld.

### 8.2. Generalisation, Calibration and Clinical Validation

Rare disease datasets create substantial risks of statistical and conceptual overfitting. Models may learn batch effects, family structure, platform artefacts or canonical-case features and perform well in internal validation while failing in patients with attenuated phenotypes, unusual mechanisms, mosaicism, dual diagnoses or underrepresented backgrounds. External validation should therefore use clinically realistic comparator groups and independent populations rather than only random partitions of a development dataset.

Performance should be reported with calibration and uncertainty measures appropriate to the intended use. High discrimination alone is insufficient if confidence is poorly calibrated or failure modes are unknown. Clinical readiness is use-case specific: variant-prioritisation algorithms, splice predictors, episignature classifiers, phenotype-matching systems and multimodal models require different validation standards and should not share a generic designation of clinically ready.

For variant interpretation, computational outputs should contribute only through appropriate ACMG/AMP- and ClinGen-aligned evidence pathways rather than as independent diagnoses. For clinically implemented assays such as selected methylation episignatures, classifier validation, quality control, comparator disorders and validated disease domain should be explicit. Research-stage multimodal models require prospective evaluation before their outputs influence classification or management.

### 8.3. Auditability, Evidence Traceability and Updateability

Several distinct concepts are frequently grouped under explainability and should not be conflated. Architecture-level interpretability concerns whether a model is intrinsically understandable; feature attribution estimates which inputs contributed to a prediction; evidence traceability identifies data, sources and analytical steps supporting an output; biological plausibility asks whether an interpretation is consistent with established mechanism; calibration describes the relationship between confidence and observed performance; and causal explanation requires evidence that the proposed mechanism produces the biological consequence. A natural-language explanation is none of these unless its claims are independently traceable and supported.

For clinical neurogenomics, the minimum requirement is auditability rather than an anthropomorphic account of model reasoning. A clinically consequential output should identify input data and quality, model version, validated domain, contributing evidence, dependencies between evidence sources, uncertainty, known failure modes and whether the output is clinical, translational or research-stage. Where applicability or evidence is insufficient, the system should support explicit abstention rather than force a prediction. Recent agentic systems such as DeepRare illustrate evidence-linked hypothesis generation, but traceable generated rationales still require source verification and clinical validation [108].

Feature attribution can assist audit but does not establish mechanism, and a biologically plausible narrative does not demonstrate causality. Updateability is equally important because gene–disease relationships, transcript annotations, population datasets and reference cohorts change. Clinical deployment therefore requires model and knowledge-base versioning, documentation of material updates, defined reanalysis triggers and mechanisms for revisiting prior interpretations. TRIPOD + AI provides a reporting framework for model development and validation, but reporting compliance is not itself evidence of clinical validity [133].

### 8.4. Governance, Privacy, Equity and Human Oversight

Multimodal neurogenomics creates governance challenges because genomic, phenotypic, facial, imaging and longitudinal clinical data may have different consent conditions and can become more identifiable when linked. Governance should address data security, purpose limitation, secondary use, data linkage, model training, retention and re-identification risk.

Equity should be treated as part of model validation. Where performance varies across ancestry, age, sex or other clinically relevant groups, these differences should be reported and incorporated into interpretation. Lack of adequate validation in a population should be represented as uncertainty rather than obscured by pooled performance metrics. Multimodal systems may also generate findings outside the original diagnostic question, requiring predefined consent, reporting and follow-up pathways.

Human oversight remains necessary when AI-derived outputs contribute to variant classification, reproductive decision-making, paediatric diagnosis, prognosis or treatment selection. Clinical accountability cannot be delegated to the model: the responsible clinician must determine whether the evidence is technically valid, biologically relevant, appropriately calibrated and applicable to the individual patient.

## 9. Future Directions

### 9.1. Mechanistic and Developmental Modelling

The next step for AI-assisted neurogenomics is not simply a more accurate classifier but models that formulate explicit, testable links between a molecular perturbation and downstream developmental consequences. Such models should be anchored to developmental brain references, patient-derived molecular measurements where available and longitudinal phenotype. Their outputs should specify what biological state is predicted to change, in which cell type or developmental window, and what observation would strengthen or refute that prediction.

Existing sequence-to-function models, single-cell atlases, trajectory methods, organoid systems, imaging models and neurophysiological analyses are components of this programme rather than a continuous validated variant-to-phenotype model. Developmental prediction should remain probabilistic, and sparse longitudinal data are a major bottleneck. Prospective validation must test calibration and trajectory prediction rather than retrospective fit alone.

### 9.2. From Mechanistic Diagnosis to Precision Therapeutics

Mechanistic diagnosis can inform therapeutic hypotheses by distinguishing correction of an initiating molecular defect from modification of a persistent downstream consequence. These are not equivalent interventions and may have different developmental windows. A splice-correcting antisense oligonucleotide, for example, targets an upstream transcript defect, whereas pathway modulation may target downstream physiology that persists after the initiating perturbation has acted.

AI-assisted models can prioritise candidate targets, estimate molecular consequences and help define measurable biomarkers, but in silico rescue is not evidence of efficacy or safety. Therapeutic prediction requires experimental confirmation, pharmacological validation and clinically meaningful endpoints. Developmental timing is particularly important in NDDs because correction of a molecular defect after a critical developmental process has passed may not reverse established circuit consequences.

### 9.3. Composable Patient-Specific Models and the Instability Twin

We use Instability Twin for a proposed computational model of an individual patient. Three properties define it. It is mechanistically constrained, meaning it represents the biology lying between a variant and a phenotype rather than mapping one onto the other statistically. It is longitudinally updateable, meaning it is revised as further data are collected from the same patient. And it can be interrogated counterfactually, meaning it can be asked what would change if a parameter were different. It is not a simulation of the brain, and no such model has been built or validated for clinical use.

The realistic near-term objective is therefore not a complete twin but a set of narrow sub-models, each with defined inputs, falsifiable predictions and independently measurable outputs. A regulatory sub-model might connect a noncoding variant to predicted enhancer activity and target gene expression, tested against measured expression in patient tissue. A cellular sub-model might position patient-derived transcriptomic data within a developmental atlas, tested against isogenic controls. A circuit sub-model might combine structural connectivity with calibrated neurophysiological parameters, tested against measured electrophysiology. Personalised virtual-brain modelling provides proof of principle for the circuit layer, but not validation of an integrated neurodevelopmental twin. Each sub-model should demonstrate calibration, external validation and incremental utility before integration is attempted because composing unvalidated components produces a model whose failures cannot be localised [120,121,122].

The term names a specific architecture rather than relabelling existing methods, and the differences are practical. A multimodal risk-prediction model returns an outcome probability without representing what happens in between, so it cannot say why the probability is what it is. A mechanistic digital twin models one physiological scale in detail and does not cross scales. A patient-specific regulatory network model reaches gene expression but stops short of cell state and circuit function. A developmental trajectory model describes movement through cell states without representing the perturbation that displaced it. An organoid perturbation model measures rather than simulates and is the substrate against which the others are tested. Two features separate the proposed architecture from all five: an explicit relationship between regulatory load and network capacity, and a developmental time axis, so that the same perturbation can be tolerated at one stage and decompensating at another. An implementation lacking either is one of the five models above and should be named accordingly.

Stated concretely, a sub-model requires the patient’s sequence data and, where available, patient-derived transcriptomic data with a stage-matched reference or imaging and electrophysiology; it is refitted as new patient data accrue or as the model version changes; it assumes the predicted molecular change is causal for the measured downstream state; it returns parameter estimates with stated uncertainty rather than outcome predictions; it is validated by concordance with an independent measurement in the same patient and by prospective correspondence with clinical course; and its intended use at present is hypothesis generation and stratification in research, not individual prediction or clinical decision-making.

## 10. Conclusions

AI-assisted neurogenomics is most useful when it moves beyond isolated prediction and helps connect genomic variation with testable molecular and developmental mechanisms. For rare and genetically enriched neurodevelopmental disorders, this requires integration of genomic, transcriptomic, epigenomic, phenotypic and, where relevant, cellular or neurophysiological evidence while preserving the distinct evidentiary status, uncertainty and biological limitations of each modality.

The load-capacity framework proposed here is intended as a hypothesis-generating structure for this integration. Regulatory load, network capacity, developmental buffering and regulatory instability should be treated as operationalisable but unvalidated constructs whose value depends on whether they can be measured independently and shown to improve explanation or prediction beyond established gene-level and simpler multimodal approaches. Regulatory instability is not synonymous with disease-associated dysregulation, and threshold-like behaviour should be demonstrated empirically rather than assumed.

Several components of this approach are already clinically or translationally relevant, including structured phenotyping, genomic reanalysis, RNA studies and selected validated episignature assays. Other elements, including patient-specific developmental models and the proposed Instability Twin, remain research concepts requiring prospective validation. Progress should therefore be judged not by architectural complexity or benchmark performance alone, but by whether models are mechanistically grounded, auditable, calibrated, externally validated and demonstrably useful for a defined clinical or research decision.

## Figures and Tables

**Table 1:** Operational vocabulary of the proposed systems-neurogenomics framework.

Construct	Operational Definition	Candidate Measurable Proxies	Principal Limitation
Regulatory network	Interacting chromatin, transcriptional, RNA-processing, signalling and other regulatory processes that influence cell state and developmental trajectory in a defined biological context.	Regulatory interactions; co-expression modules; chromatin accessibility; enhancer–promoter relationships; transcription-factor occupancy; pathway connectivity.	Network definitions are context- and data-dependent and may not correspond directly to causal biological interactions.
Developmental context	The cell type, developmental stage, anatomical location and physiological state in which a molecular perturbation acts.	Cell-type-specific expression; chromatin state; developmental atlases; spatial expression; longitudinal phenotype.	Reference datasets may not represent the patient’s relevant tissue, developmental stage or state.
Regulatory load	A latent construct describing the effective perturbational burden imposed by a genomic or molecular alteration within a specified biological context.	Magnitude of molecular effect; breadth of affected targets; persistence; developmental timing; independently defined regulatory reach or network position.	No validated composite measure exists; should not be inferred from clinical severity.
Network capacity	Properties of the recipient biological system that may constrain propagation of a perturbation or preserve functional fidelity.	Regulatory redundancy; compensatory paralogue/pathway activity; feedback architecture; alternative regulatory routes; experimentally measured recovery after perturbation.	Capacity is context-dependent and difficult to measure independently; mild phenotype is not evidence of high capacity.
Developmental buffering	Observed attenuation of perturbational effects sufficient to preserve an expected developmental state or trajectory.	Preserved cell-state fidelity; preserved differentiation trajectory; recovery after perturbation; reduced effect despite comparable primary perturbation.	Must be distinguished from molecular compensation and should not be inferred retrospectively from outcome alone.
Regulatory network instability	Reduced stability of the maintenance, transition or recovery of a regulatory or cellular state.	Excess residual biological variability; altered transition probabilities; increased trajectory dispersion; impaired recovery after perturbation; reduced state fidelity.	Disease-associated dysregulation alone does not establish instability; appropriate dynamical measurements are required.
Transcriptional variability/noise	Residual biological cell-to-cell expression variability after relevant technical and biological sources of variation have been accounted for.	Residual expression variance; transcriptional dispersion; variability within appropriately matched cell states.	Raw expression variance conflates technical noise, cell composition, developmental heterogeneity and physiological variation.
Threshold-like response	An empirically supported nonlinear transition or change point in a prespecified molecular, cellular or developmental outcome.	Change-point analysis; model comparison against continuous and smoothly nonlinear alternatives.	Should not be assumed from categorical phenotype or sigmoid behaviour; may not exist in a given system.
Instability Twin	A proposed patient-specific, longitudinally updateable, mechanistically constrained and counterfactually interrogable computational architecture integrating validated sub-models across relevant biological scales.	Patient-specific regulatory, cellular, developmental or circuit sub-models with prospectively testable outputs.	Conceptual/research-stage; no complete clinically validated Instability Twin currently exists.

**Table 2:** AI approaches commonly used in neurogenomics.

AI Approach	Typical Input	Output	Representative Tools	Key References
Classical supervised learning	Labelled variants, methylation profiles, phenotype descriptors	Pathogenicity score, disease-class probability, episignature assignment	REVEL, ClinPred, SVM-based episignature classifiers	[34,35,37,38,39,40,41]
Unsupervised and self-supervised learning	Methylation, transcriptomic or single-cell feature matrices	Clusters, low-dimensional embeddings, molecular subgroups, trajectory structure	PCA, t-SNE, UMAP; episignature cohort subgroup discovery	[34,35,37]
Deep learning (sequence and image models)	DNA/RNA sequence, protein sequence, clinical images, spatial transcriptomic data	Predicted splice effect, missense impact, regulatory consequence, image-derived phenotype score	SpliceAI, AlphaMissense, AlphaGenome, GestaltMatcher	[23,26,38,39,40,41,42,43,44,45,46,47,48,49]
Foundation models (pre-trained, transferable)	Large-scale sequence, protein or cell-state corpora	Biological embeddings; zero-shot or fine-tuned task predictions	ESM-2, Nucleotide Transformer, Geneformer	[34,35,36]
Knowledge graphs and ontologies	HPO terms, gene–disease relationships, pathway annotations	Semantic similarity scores, phenotype-driven gene rankings, pathway connections	Phenomizer, Exomiser, HPO-driven prioritisation pipelines	[50,51,52,53,54,55,56,57,58,59,60,61,62,63]

REVEL, Rare Exome Variant Ensemble Leaner; SMV, Support Vector Machine; HPO, Human Phenotype Ontology; ESM-2, Evolutionary Scale Modeling 2; PCA, principal component analysis; SVM, support vector machine; t-SNE, t-distributed stochastic neighbour embedding; UMAP, uniform manifold approximation and projection.

**Table 3:** Representative tools and concepts across the AI-assisted neurogenomics workflow.

Layer	Representative Tools/ Approaches	Clinical Role and Principal Limitation	Readiness/Key References
Variant prioritisation	REVEL, CADD, AlphaMissense, popEVE, pLI/LOEUF, TALOS	Prioritises candidates and may support computational evidence; scores do not independently establish pathogenicity or mechanism.	Clinical/near-clinical [37,39,40,49,50,51,52,53,68,69,70,71,72,73,74]
Splicing and RNA processing	SpliceAI, Pangolin, AbSplice, FexSplice; RNA-seq	Predicts splice effects and directs RNA confirmation. Informative RNA may provide functional evidence; negative RNA may be non-informative in the wrong tissue/transcript.	Clinical/near-clinical to translational [25,27,28,77,78,79]
Protein structure and function	AlphaFold, ColabFold, Missense3D, ESM-2	Refines domain/interface hypotheses and prioritises functional testing; predicted structural effects do not establish in vivo mechanism.	Translational [80,81,82,83,84,85,86,87]
Regulatory genomics	AlphaGenome, EPInformer, TF-binding models	Prioritises noncoding candidates and predicts sequence-to-function effects; patient-specific and brain-relevant validation remain limited.	Emerging [43,44,75,76,88,89]
Clinical phenomics	Exomiser, Phenomizer, PhenoBERT, PhenoTagger, HPO-based tools	Supports phenotype-gene concordance and ranking; depends on phenotype quality, age and knowledge-base completeness.	Clinical/near-clinical [50,51,52,53,54,56,57,58,59,60,61,62,63,90,91,92,93,94]
Facial/dysmorphology phenomics	GestaltMatcher, DeepGestalt, Face2Gene, FaceMatch, Image2Gene	Adjunctive syndrome prioritisation; performance depends on ancestry, age, consent, governance and training domain.	Clinical adjunct/near-clinical [38,39,40,41,42,95,96]
Episignature diagnostics	Disorder-specific methylation classifiers; MVP pipelines	May provide functional support for validated signatures and selected VUS interpretation; blood is a surrogate tissue, and results are classifier-domain-dependent.	Clinical/near-clinical for validated signatures [22,24,97,98,99,100,101,102,103,104,105,106]
Single-cell and spatial biology	Brain atlases, STAB2, ZEBRA, Geneformer, spatial models	Defines vulnerable cell types, developmental windows and anatomical context; currently not direct clinical variant-classification evidence.	Emerging [15,90,107,108,109,110,111,112]
Developmental trajectory modelling	iPSC/organoids, RNA velocity, TarDis	Tests timing and cellular trajectory divergence; limited by model-system fidelity and differentiation variability.	Translational/emerging [2,19,20,22,107]
Integrative diagnostic reasoning	Agentic LLM systems; multi-agent evidence integration	Synthesises heterogeneous evidence and ranks hypotheses; generated rationales require source verification and calibration.	Emerging [108]
Therapeutic target discovery	Pathway models, drug-target predictors, ASO design, perturbation screens	Generates therapeutic hypotheses; in silico prioritisation is not evidence of efficacy, safety or clinical utility.	Translational/emerging [109,110]

Readiness does not imply equivalent evidentiary weight. Clinical/near-clinical denotes externally validated methods used or readily implementable in diagnostic workflows; translational denotes substantial research support without broad prospective validation; emerging denotes early-stage or restricted-dataset approaches; conceptual denotes proposed architectures without patient-level validation. Computational predictions generally prioritise or support hypotheses rather than provide autonomous clinical classifications. HPO, Human Phenotype Ontology; MVP, methylation variant pathogenicity.

## Data Availability

No new data were created or analysed in this study. Data sharing is not applicable to this article.

## Abbreviations

The following abbreviations are used in this manuscript:

REVEL	Rare Exome Variant Ensemble Learner
CADD	Combined Annotation-Dependent Depletion
popEVE	population-calibrated Evolutionary Variational model Ensemble
pLI	probability of being loss-of-function intolerant
LOEUF	Loss-of-function Observed/Expected Upper bound Fraction
ESM2	Evolutionary Scale Modeling 2
TF-binding models	transcription factor-binding models
HPO	Human Phenome Ontology
MVP pipelines	Methylation variant pathogenicity pipelines
ZEBRA	Zebrafish Expression Brain Atlas
iPSC	induced pluripotential stem cell
TarDis	Targeted Disentanglement; LLM, large Language model
ASO	antisense oligonucleotide
